# A comparison of internal model validation methods for multifactor dimensionality reduction in the case of genetic heterogeneity

**DOI:** 10.1186/1756-0500-5-623

**Published:** 2012-11-05

**Authors:** Jeffrey J Gory, Holly C Sweeney, David M Reif, Alison A Motsinger-Reif

**Affiliations:** 1 Bioinformatics Research Center, Department of Statistics, North Carolina State University, Raleigh, NC, 27695, USA

## Abstract

**Background:**

Determining the genes responsible for certain human traits can be challenging when the underlying genetic model takes a complicated form such as heterogeneity (in which different genetic models can result in the same trait) or epistasis (in which genes interact with other genes and the environment). Multifactor Dimensionality Reduction (MDR) is a widely used method that effectively detects epistasis; however, it does not perform well in the presence of heterogeneity partly due to its reliance on cross-validation for internal model validation. Cross-validation allows for only one “best” model and is therefore inadequate when more than one model could cause the same trait. We hypothesize that another internal model validation method known as a three-way split will be better at detecting heterogeneity models.

**Results:**

In this study, we test this hypothesis by performing a simulation study to compare the performance of MDR to detect models of heterogeneity with the two different internal model validation techniques. We simulated a range of disease models with both main effects and gene-gene interactions with a range of effect sizes. We assessed the performance of each method using a range of definitions of power.

**Conclusions:**

Overall, the power of MDR to detect heterogeneity models was relatively poor, especially under more conservative (strict) definitions of power. While the overall power was low, our results show that the cross-validation approach greatly outperformed the three-way split approach in detecting heterogeneity. This would motivate using cross-validation with MDR in studies where heterogeneity might be present. These results also emphasize the challenge of detecting heterogeneity models and the need for further methods development.

## Background

An important problem in human genetics is the challenge of identifying polymorphisms that are associated with high disease risk. This task can be difficult because the underlying genetic models of many common human diseases, such as heart disease and Type II diabetes, are complex in their genetic etiology [1]. For instance, there can be gene-gene interactions (known as epistasis) or multiple genotypes that result in the same phenotype (known as genetic heterogeneity) [2]. Epistasis creates a challenge for traditional analytical approaches and these challenges in feature selection and parameter estimation for epistasis models have been previously discussed in the literature [2-4].

To address these problems, a number of new approaches have been developed to try to detect interactions [5,6]. Recent approaches take a broad range of computational approaches to detect and characterize epistasis, including exhaustive search techniques [7,8], two-stage screening approaches [9], Bayesian approaches [10], evolutionary algorithms [11], tree-based approaches [12], etc. Each of these approaches has advantages and disadvantages for a range of genetic etiologies and dataset sizes [13-15]. Recently, a hand-curated database of all reported interactions in human genetics documented the methods used to discover these interactions [16]. In the reported interactions, about 37% were detected using new machine-learning methods (as opposed to traditional statistical techniques such as regression, analysis of variance, etc.). Of those, Multifactor Dimensionality Reduction (MDR) [7], was used the most (in 35% of the studies using new methods, representing a total use in 13% of the studies reporting interactions). This widespread use motivates the further investigation of practical implementation issues with this method.

MDR is a nonparametric procedure that reduces the dimensionality of the data by classifying each genotype as either high-risk or low-risk and then uses internal model validation, typically either five-fold or ten-fold cross-validation (CV), to select the best model [17]. MDR with CV has become common in genetic epidemiology and has successfully found interactions in both simulated and real data related to such diseases as schizophrenia, sporadic breast cancer, multiple sclerosis, and atrial fibrillation. A recent review of the MDR approach and its extension and application can be found in [18].

One drawback of MDR with CV is that it is computationally intensive because it performs an exhaustive search of all possible combinations of factors. Further, the use of m-fold CV for internal model validation requires that the MDR algorithm be executed m times for each possible combination, which adds to the computation time. To help reduce the required computation an alternative internal model validation method, the three-way split (3WS), has been incorporated into the MDR algorithm [19]. MDR with 3WS has been shown to be significantly faster than MDR with CV and it does not result in a significant loss in the ability to detect interactions [19]. MDR with 3WS does tend to fit a larger model than MDR with CV, so false positives are more common with 3WS and a pruning procedure may need to be employed if Type I error is to be avoided [19].

Another drawback of MDR is that it performs poorly in the presence of genetic heterogeneity [20,21]. Genetic heterogeneity (where more than one model underlies disease risk) is a problem for a number of machine learning methods [22]. There are several potential reasons that MDR performs poorly in the presence of heterogeneity, as discussed in these previous studies [20,21]. The use of cross-validation is one potential reason – since the usual application of MDR is to pick a single best model, if there are two competing models such as is the case in heterogeneity situations, no single model may emerge as consistently chosen, resulting in a low cross-validation consistency for all models. It is possible that MDR with 3WS could perform better than MDR with CV in such situations because the 3WS algorithm uses a different approach to screen potential models (allowing multiple models to be passed along at each stage) and tends to fit a larger model. To our knowledge, no study has been done to investigate the power of MDR with 3WS in the presence of genetic heterogeneity.

The purpose of the present study is to compare the effectiveness of MDR with CV to that of MDR with 3WS in situations wherein genetic heterogeneity is present. This is accomplished through simulating genetic data exhibiting heterogeneity and evaluating the success of the two internal model validation methods at identifying the correct underlying models. It is necessary to use simulated data because we must know the true underlying model in order to assess the accuracy of the predicted model and such information is not known with real data.

## Methods

### Multifactor Dimensionality Reduction (MDR)

MDR is a widely used data mining technique that performs an exhaustive search of all possible genes and combinations of genes to find the best model for a certain genetic trait [23]. It is able to accommodate more complex genetic traits that involve gene-gene and gene-environment interactions [7]. MDR uses combinatorial data reduction techniques to collapse the high dimensions of complex genetic data into just one dimension with two levels (high-risk and low-risk) [7]. MDR is nonparametric as no assumptions about the underlying statistical distribution or genetic models are made. For the following description consider a set of genetic data of sample size N (with n_1_ cases and n_0_ controls) for which the genotypes at K loci are known and it is believed that the largest interaction involves k terms.

The first step in the MDR algorithm is to enumerate all possible combinations of k loci. For each combination of loci the number of cases and controls are counted for every possible combination of genotypes. For genes with two possible alleles each locus has three possible genotypes, so the data can be classified into 3^k^ genotypic combinations. We will refer to each such combination as a multifactor class. The ratio of cases to controls is calculated for each multifactor class using the sample data and this value is used to classify each multifactor class as either high-risk or low-risk. In the case of balanced data, meaning data with an equal number of cases and controls, the multifactor classes with a case-to-control ratio exceeding one are considered high-risk while those with a ratio below one are considered low-risk. In general the threshold is n_1_/n_0_. This high-risk/low-risk parameterization serves to reduce the high dimensionality of the data.

**Figure 1:**
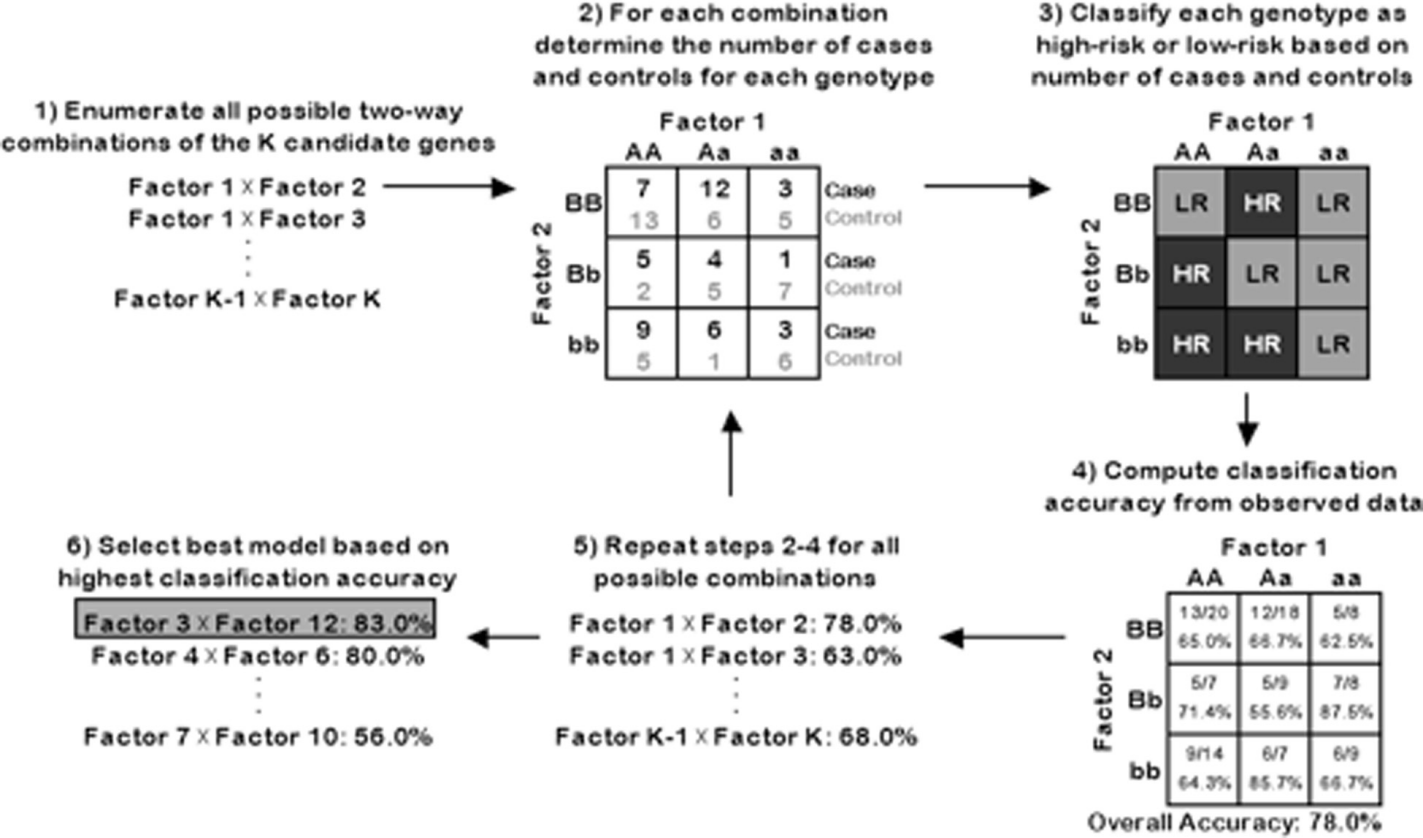
Workflow of the MDR process.

After each multifactor class is categorized as high-risk or low-risk, the observed data are compared to the resulting model to determine what proportion of the observations are classified correctly. The goal is to find the model that minimizes the misclassification rate. When the sample does not include an equal number of cases and controls, balanced accuracy, the mean of sensitivity and specificity, is used [24]. Using this criterion a best model is found for each size model up to k loci. To avoid over-fitting it is common to use an internal model validation procedure (either cross-validation or three-way split) to select the overall best model. The statistical significance of the selected model’s prediction accuracy can be evaluated through permutation testing. Figure 1 illustrates the general MDR algorithm for models of size k=2.

### Cross-validation (CV)

**Figure 2:**
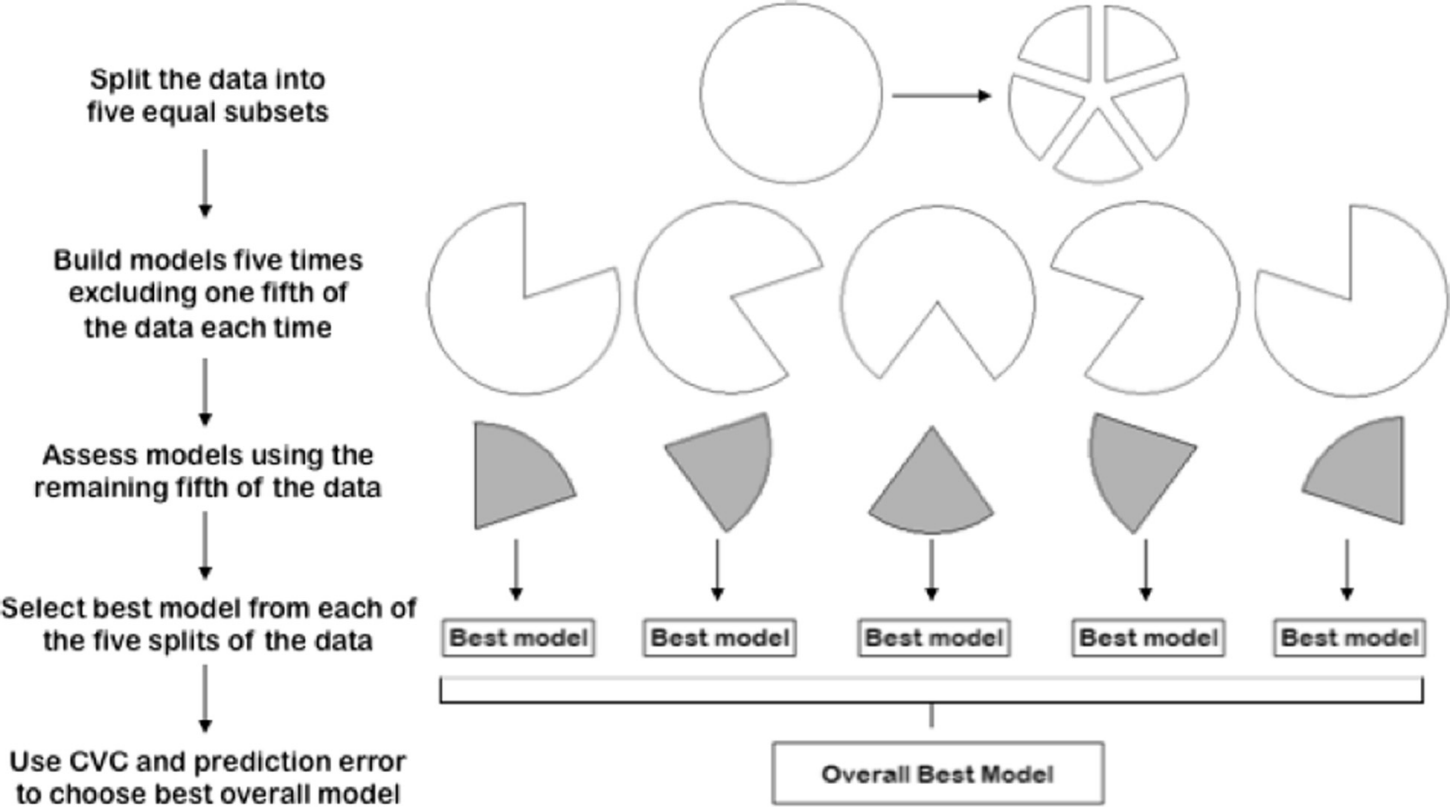
Workflow of the five-fold cross-validation process.

CV is the internal model validation method most commonly used with MDR. Before running the MDR algorithm on any data the full dataset is split into m equal intervals. One of these intervals is considered the testing set while the other m-1 intervals make up the training set. MDR is run on the training data for each of the m possible splits of the data. That is, for each possible combination of k loci MDR is run m times with a different interval being excluded from the analysis each time. After the high-risk and low-risk categories are determined using the training set, the predictive capability of the resulting model is determined using the testing set. For each split of the data and each size of interaction the model that maximizes the prediction accuracy, meaning the one that minimizes the misclassification rate for the testing data, is considered the best model for that size interaction. This process is illustrated in Figure 2.

The number of times that a particular model is identified as the best model across the m subsets of the data is known as the cross-validation consistency. The model chosen as the best overall model is the one that has both the highest prediction accuracy and the highest cross-validation consistency. If the model that maximizes prediction accuracy is different than the model that maximizes cross-validation consistency, then the more parsimonious model is chosen [21]. CV is most commonly employed with either five or ten equal splits of the data. It has been shown that five and ten splits yield similar results [17], so this study utilizes five splits of the data to lessen computing time.

### Three-way split (3WS)

3WS is an internal model validation method that has only recently been implemented with MDR. For this procedure, the full dataset is randomly split into three parts: a training set to build initial models, a testing set to narrow the list of potential models, and a validation set to choose the best model and assess its predictive capability. It has been shown that the proportion of the data included in each split does not make a major difference in the resulting model, but the optimal split, and the one we use, is a 2:2:1 ratio [19]. MDR is run using each of these three sets with every possible combination of up to k loci considered with the training set, a subset of these possible combinations considered with the testing set, and only the top few models considered with the validation set. The three splits of the data can be considered independent of one another and balanced accuracy can be calculated for each combination of loci to determine the best model. This method is much more computationally efficient than CV because the MDR algorithm is carried out fewer times and fewer models are considered each time.

**Figure 3:**
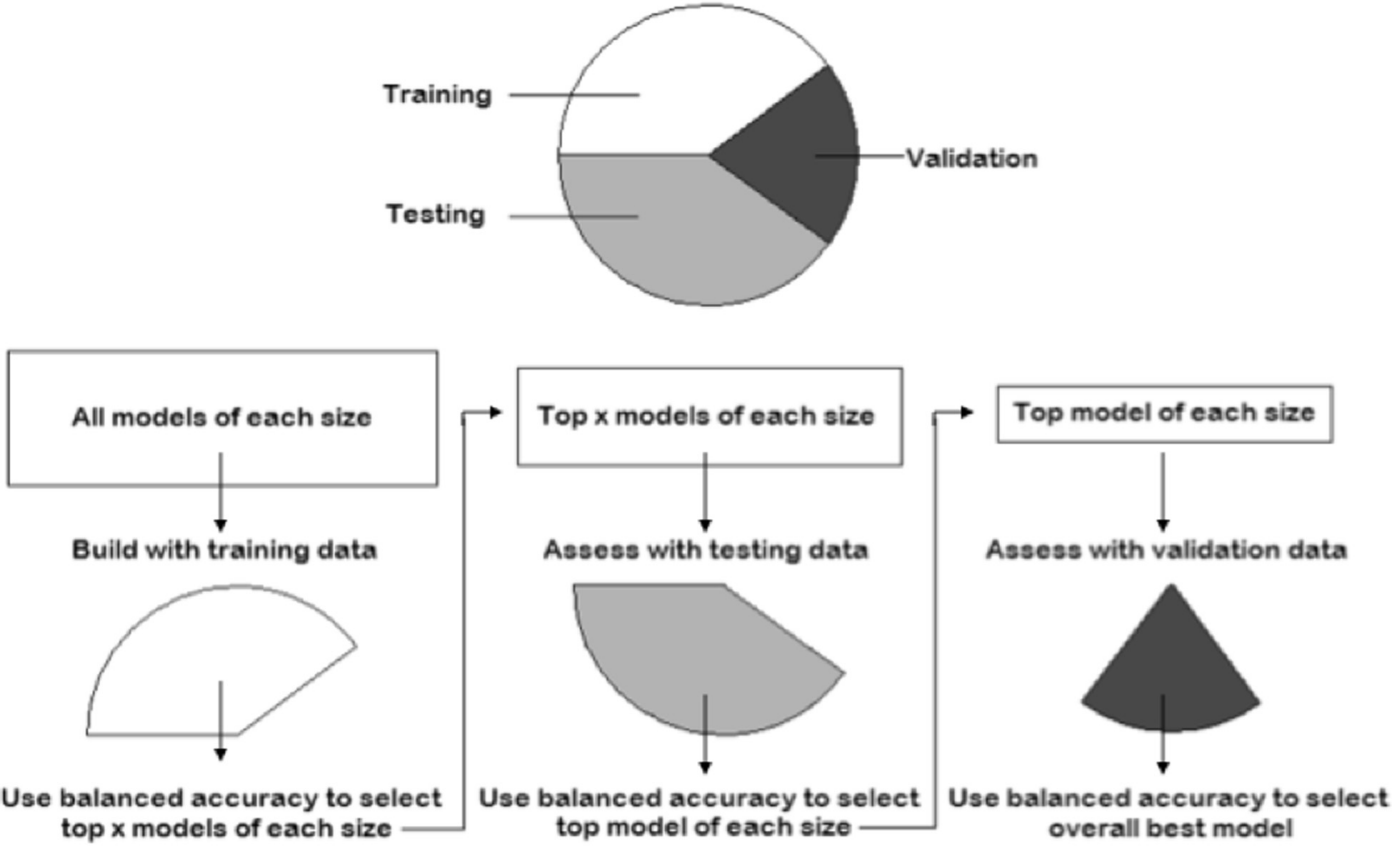
Workflow of the three-way split approach.

When MDR is performed on the training set all possible combinations of loci for each size combination up to size k are considered. The top x models for each size are chosen based on balanced accuracy and these models move on to the testing set. The value of x is arbitrary and is chosen by the user. A common practice, and the one we use in our analysis, is to set x equal to K, the total number of loci being considered. This “rule of thumb” was proposed based on the results of a parameter sweep comparing the performance of MDR with different splits of the data [19]. MDR is carried out again on the top x models for each size and only the model with the greatest balanced accuracy for each size moves on to the validation set. In the validation set MDR is carried out a final time on the top model for each size and the model with the greatest balanced accuracy is chosen as the overall best model. This process is illustrated in Figure 3.

### Data simulation

**Figure 4:**
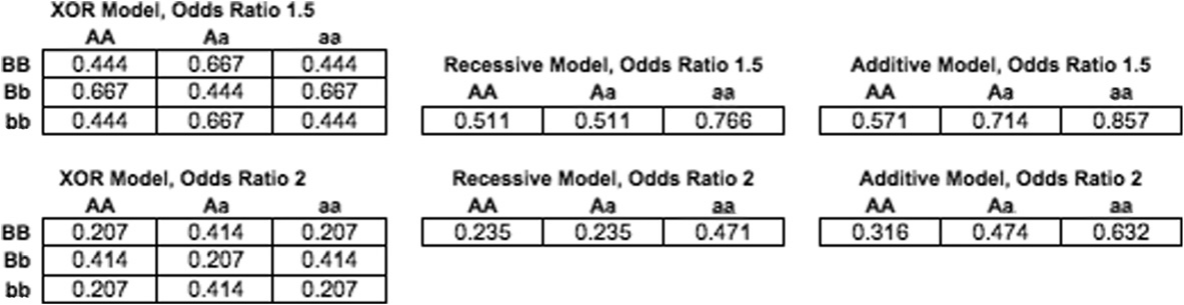
Penetrance tables for the models simulated.

To determine if MDR with 3WS can better detect genetic heterogeneity than MDR with CV we performed a simulation-based study so that we could calculate the empirical power for both methods (since theoretical power calculations are not possible with MDR). Factors of interest considered were the number of loci in the true disease model, the structure of the true model, the odds ratio, and the level of heterogeneity. In particular, genetic heterogeneity models consisting of two one-locus models or two two-locus models were simulated. The one-locus models involved additive or recessive effects while the two-locus models followed an XOR model, which is an epistatic model that has been previously discussed in the literature [25]. Since the heterogeneity models combine two models, we ultimately tested MDR’s capability to detect two-locus and four-locus models. The levels of heterogeneity considered were 50/50, meaning the two disease models going into the simulation carried equal weight, and 25/75, meaning one disease model carried more weight than the other. The odds ratios considered were 1.5 and 2. Each of the models had a heritability (h^2^) of .05 which is a very low genetic signal compared to many genetic diseases. The penetrance tables for the models simulated are shown in Figure 4, where penetrance is the probability of disease given a particular genotype combination.

We simulated a total of 21 genetic heterogeneity models. Table 1 shows the type of model, level of heterogeneity, and odds ratio for each model simulated. There were 14 two-locus models that varied by model type (additive or recessive), level of heterogeneity (50/50 or 25/75), and odds ratios (1.5 or 2). The remaining seven simulations involved XOR models that varied by level of heterogeneity (50/50 or 25/75) and odds ratios (1.5 or 2). Using the GenomeSIM software [26], 100 datasets of sample size 1000 were generated for each of the 21 models. Each dataset includes 500 cases and 500 controls so the data are balanced. Each dataset also has a total of 25 loci meaning either 23 or 21 noise loci to go along with the two or four disease loci depending on the size of the true model. Although most candidate gene studies include more than 25 total loci it has been previously shown that additional noise loci do not affect the power of MDR [27], so fewer loci were included to reduce computing time and make the simulation study feasible.

**Table 1:** Summary of genetic models simulated

				**First model**		**Second model**	
**Simulation**	**Disease loci**	**Model type**	**Level of heterogeneity**	**Odds ratio**	**Contribution**	**Odds ratio**	**Contribution**
1	2	additive	25/75	1.5	25%	1.5	75%
2	2	additive	25/75	2	25%	2	75%
3	2	additive	25/75	1.5	25%	2	75%
4	2	additive	25/75	2	25%	1.5	75%
5	2	additive	50/50	1.5	50%	1.5	50%
6	2	additive	50/50	2	50%	2	50%
7	2	additive	50/50	1.5	50%	2	50%
8	2	recessive	25/75	1.5	25%	1.5	75%
9	2	recessive	25/75	2	25%	2	75%
10	2	recessive	25/75	1.5	25%	2	75%
11	2	recessive	25/75	2	25%	1.5	75%
12	2	recessive	50/50	1.5	50%	1.5	50%
13	2	recessive	50/50	2	50%	2	50%
14	2	recessive	50/50	1.5	50%	2	50%
15	4	XOR	25/75	1.5	25%	1.5	75%
16	4	XOR	25/75	2	25%	2	75%
17	4	XOR	25/75	1.5	25%	2	75%
18	4	XOR	25/75	2	25%	1.5	75%
19	4	XOR	50/50	1.5	50%	1.5	50%
20	4	XOR	50/50	2	50%	2	50%
21	4	XOR	50/50	1.5	50%	2	50%

### Analysis

All 100 datasets for each of the 21 simulations were analyzed using MDR with five-fold CV and MDR with 3WS. This was done using the MDR package available for the statistical software R [28,29]. For MDR with 3WS we used the default split of 2:2:1 (train:test:validate) and a value of x=25 (the total number of loci in each dataset) to allow 25 models to pass from the training set to the testing set. For both methods MDR considered models of size k=1,2 for the two-locus models and k=1,2,3,4 for the four-locus models.

We collected the output from these MDR procedures to assess the accuracy of the final models. Power was calculated as the percentage of times out of the 100 datasets for each simulation that the final model met some specified criterion. We initially computed a conservative estimate of power for which this criterion was that the final predicted model included all of the true disease loci and no false positive loci. It was immediately apparent that both methods did a poor job finding the entire correct model. We therefore defined several more liberal types of power to assess how often each method found at least one of the two models included in the heterogeneity model. For the power labeled mod1 a trial was considered a success if at least the locus or loci of the first of the two models contributing to the heterogeneity model was included in the final predicted model. For the power labeled onlymod1 the requirement was that the final predicted model be exactly the first of the two simulated models contributing to the overall model with no additional loci included. The power definitions mod2 and onlymod2 are analogous to mod1 and onlymod1, but for the second of the two models. We also defined a power, labeled nofalse, that considered a trial a success if the predicted model included any number of correct loci and no false positive loci.

Differences between the performances of the two internal model validation methods were tested using an analysis of variance (ANOVA), implemented in SASv9.2 [30].

## Results and discussion

**Figure 5:**
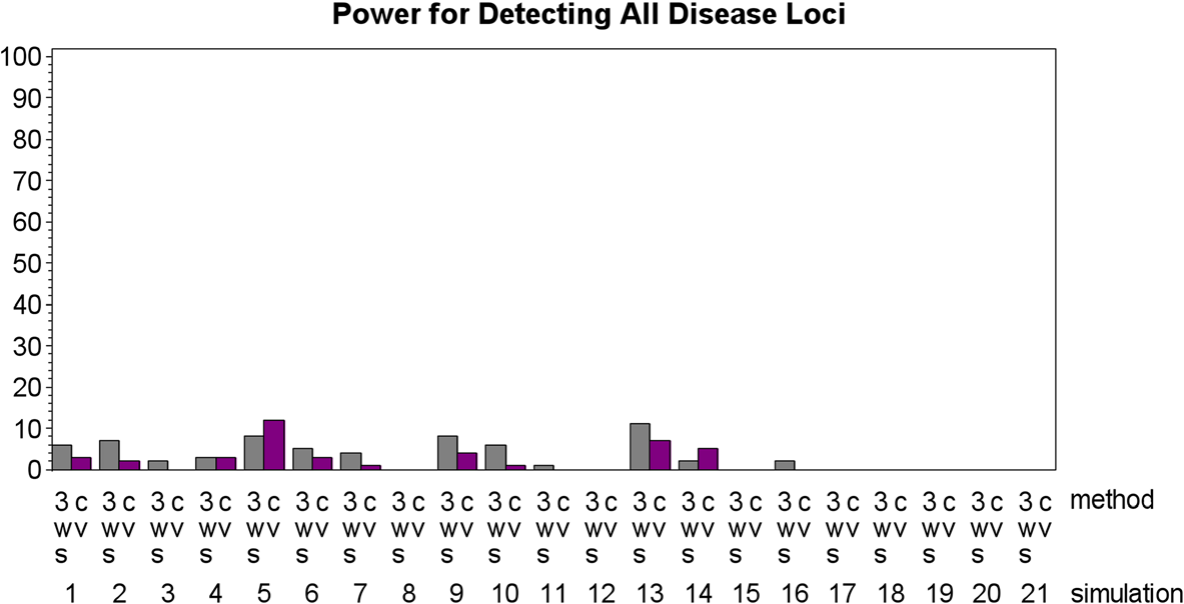
Power results for both the three-way split (3WS) and cross-validation (CV) implementations of MDR where power is defined as the percentage of times that both models were identified (with no false positive and no false negative loci).

MDR was rarely able to detect the true disease model for both the two-locus and four-locus heterogeneity models regardless of whether it was implemented with 3WS or five-fold CV. This is an expected result given previous studies that have examined the power of MDR to detect heterogeneity [20,21]. In fact, for all 21 simulations the most often that a single method found all of the true disease loci across the 100 simulated datasets was twelve. This poor conservative power is illustrated in Figure 5. Because both 3WS and five-fold CV had such poor conservative power, neither proved to be significantly better than the other by this measure (p-value = .1637) when an analysis of variance (ANOVA) was run on the results.

**Figure 6:**
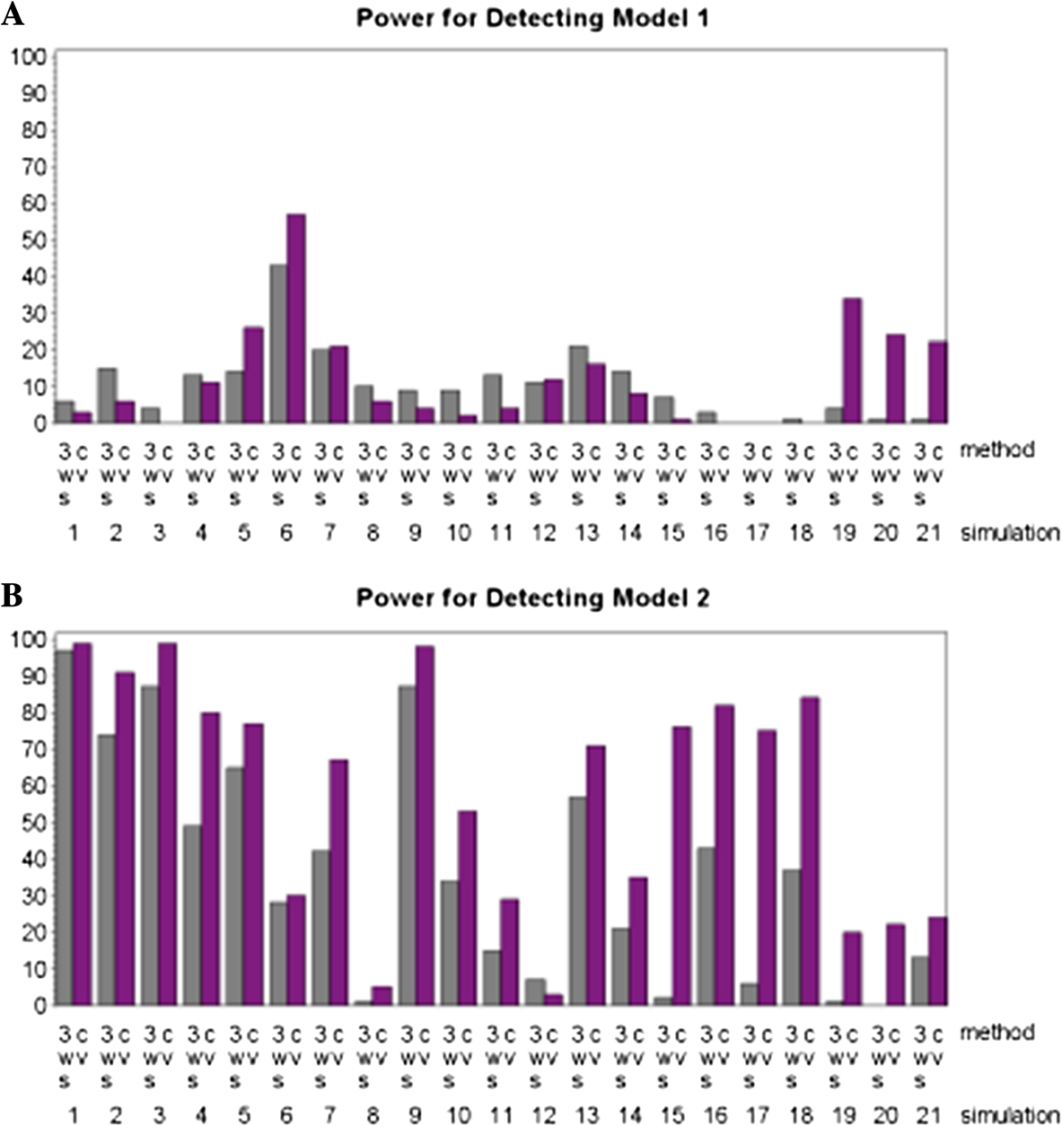
**Power results for both the three-way split (3WS) and cross-validation (CV) implementations of MDR where power is defined as the percentage of times that one of the underlying models was identified (with no false negative loci but allowing false positive loci). **The results for Model 1 are shown in **A**, and the results for Model 2 are shown in **B**.

Since the conservative power estimates did not provide much information as to which internal model validation method has better performance, 3WS and CV were compared using more liberal estimates of power. These alternative forms of power will be referred to as mod1, mod2, onlymod1, onlymod2, and nofalse. The criteria for mod1 and mod2 was that the final predicted model include all of the true disease loci in either the first or second of the two models contributing to the overall heterogeneity model. This is not as stringent as the conservative power that required all the true disease loci from both of the contributing models to be included in the final predicted model. By easing back the requirement for the method to be considered a success we saw an improvement in performance and the emergence of differences between the two methods. This approach is similar to previous studies that considered heterogeneity [20,21]. In particular, power sharply improved for both methods when looking at mod2. A much smaller improvement was seen for mod1. This disparity is due largely to the fact that in half of the simulations the first model only carries 25% of the weight whereas the second model contributes 75% to the overall model. This makes the first model much more difficult to detect in these situations and results in the lower success rate in terms of identifying the first model versus identifying the second. Figure 6 summarizes these results.

**Figure 7:**
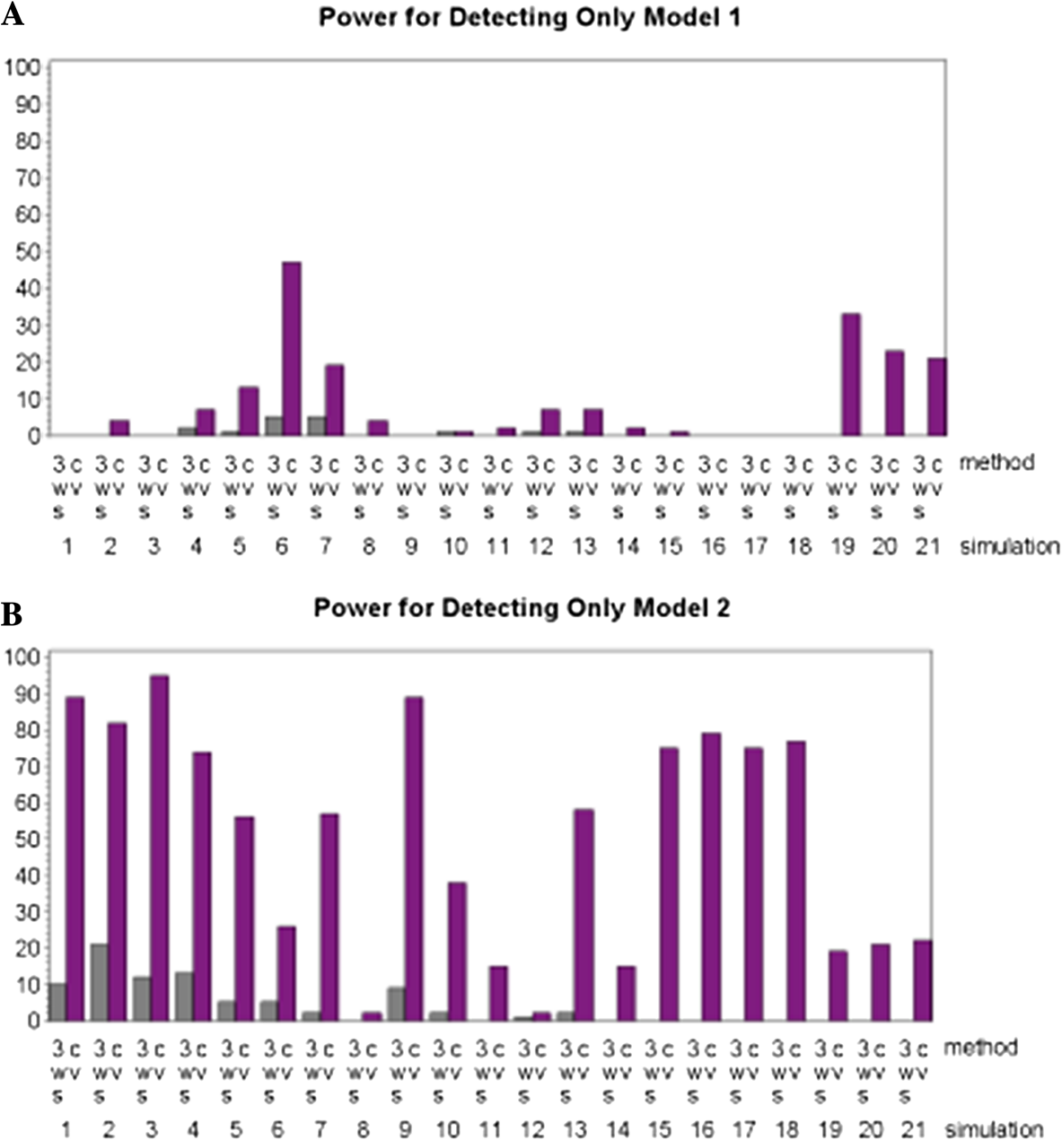
**Power results for both the three-way split (3WS) and cross-validation (CV) implementations of MDR where power is defined as the percentage of times that one of the underlying models was identified (with no false positive and no false negative loci). **The results for Model 1 are shown in **A**, and the results for Model 2 are shown in **B**.

Two more stringent definitions of power that accounted for the inclusion of false positives in the final predicted model, onlymod1 and onlymod2, saw similar improvements in power (when compared to conservative power) for MDR implemented with CV. These definitions of power required that exactly one of the two contributing models be identified with no additional loci included in the final predicted model. For MDR implemented with CV there was a drastic improvement in terms of finding the second model and a minor improvement in terms of detecting the first model. However, MDR implemented with 3WS had very little success detecting either model. Figure 7 summarizes these results. Ultimately, MDR implemented with CV saw little drop off in performance from mod1 to onlymod1 and from mod2 to onlymod2 while MDR implemented with 3WS saw a severe decline in performance when false positives were counted against the model. This suggests that MDR implemented with 3WS has a higher rate of false positives.

**Figure 8:**
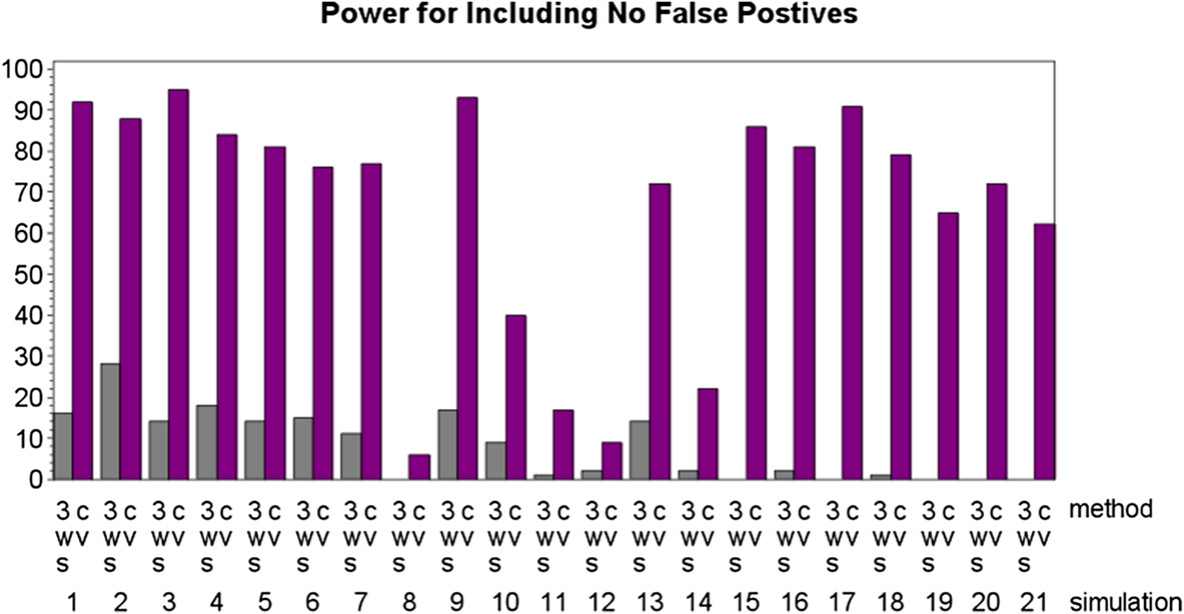
**Power results for both the three-way split (3WS) and cross-validation (CV) implementations of MDR where power is defined as the percentage of times that any of the correct loci were identified (allowing false negative loci but not false positive loci)**.

In fact, MDR implemented with 3WS tends to choose a larger final model than MDR implemented with CV. For the two-locus heterogeneity models the mean size of the final predicted model was 1.89 for 3WS (mode of 2) and 1.21 for CV (mode of 1). For the four-locus heterogeneity models the mean size of the final predicted model was 3.99 for 3WS (mode of 4) and 1.84 for CV (mode of 2). Since 3WS tended to choose the largest possible final model, it had poorer power in terms of producing final models that included exactly one of the two contributing models with no additional loci. It also had a tendency to produce fewer models with no false positives (power labeled nofalse). For all 21 simulations, MDR implemented with CV produced a final predicted model with no false positives in at least six more of the 100 datasets than MDR implemented with 3WS did. In most cases the difference between the two methods was much greater with the disparity between number of datasets yielding a predicted model with no false positives getting as high as 91. This is illustrated in Figure 8. This measure did not take into account how many true disease loci were identified, only that no loci were incorrectly identified, so while it shows that 3WS tends to include more false positives it says nothing about the rate of false negatives.

For both the two-locus and four-locus heterogeneity models, MDR implemented with CV tended to outperform MDR implemented with 3WS based on the more liberal definitions of power. Statistical significance (at α = .05) was achieved for mod2 (p-value=.0056), onlymod1 (p-value=.0012), onlymod2 (p-value >.0001), and nofalse (p-value > .0001). The greatest differences in performance were seen with onlymod2 and nofalse where CV had extremely high power while 3WS had minimal power. The only liberal definition of power that did not see a significant difference was mod1. This lack of significance resulted more from the poor performance of MDR implemented with CV than from the strong performance of MDR implemented with 3WS. Many of the models that needed to be identified to be considered a success for this type of power contributed only 25% to the overall heterogeneity model, so they were extremely hard to detect. While the performance of MDR implemented with CV was about the same for mod1 as for onlymod1, there was a significant difference between CV and 3WS based on onlymod1 because MDR implemented with 3WS almost never identified the first model without including any additional loci.

The results of the ANOVA analysis to evaluate the results of the simulations experiment are shown in Table 2. In general, as expected, the simulation factors also had an effect on MDR’s performance. For conservative power, OR and model type (XOR, additive, or recessive) significantly impacted the performance of MDR. For the more liberal definitions of power, OR generally did not have a significant impact on performance, but level of heterogeneity and model type generally did. Level of heterogeneity had the biggest impact on power and was found to be statistically significant (at α = .05) for all liberal types of power except nofalse. Model type was not significant for onlymod1 or nofalse, but it was for everything else. Odds ratio impacted nofalse, but not any of the other liberal powers.

**Table 2:** P-values from the ANOVA analysis of the simulation results

**Effect**	**Conservative**	**mod1**	**onlymod1**	**mod2**	**onlymod2**	**nofalse**
internal model validation method	0.1637	0.5136	0.0012	0.0056	< .0001	<.0001
level of heterogeneity	0.2482	< .0001	0.0003	0.0005	0.001	0.0733
model type	0.0006	0.0147	0.1672	0.0004	0.0109	0.155
odds ratio (OR)	0.0444	0.2025	0.7075	0.18	0.3708	0.0003

In terms of computing time, MDR implemented with 3WS was approximately five times faster than MDR implemented with CV. This is consistent with results published by Winham et al. [8]. The majority of the computation time is spent classifying all possible combinations of loci as either high-risk or low-risk and calculating a balanced accuracy estimate for all these combinations in the training set. This process is done only once with 3WS and five times for five-fold CV, so 3WS is theoretically five times faster than five-fold CV. The difference in efficiency also depends on many other factors such as sample size and the total number of loci [19].

## Conclusion

While MDR implemented with CV has been effective at detecting disease models exhibiting epistasis, it has been shown to have a dramatic decrease in power in the presence of genetic heterogeneity [20,21]. Recently, an alternative internal model validation method, the 3WS, has been shown to have roughly the same power as CV for detecting standard epistatic models when implemented with MDR [19]. The main conclusion to draw from this study is that MDR implemented with 3WS not only fails to detect disease models exhibiting genetic heterogeneity better than MDR implemented with CV, but by some measures it performs significantly worse. While we recognize that the current study does not provide solutions for improving detection of heterogeneity, we do hope this study provides important practical guidance when choosing an internal model validation approach.

Both 3WS and CV perform extremely poorly in terms of detecting the full heterogeneity model. Neither method did significantly better than the other in this respect, but neither performed well enough to have any practical utility. Looking at more liberal definitions of power, for which it was considered a success if MDR detected one of the two models contributing to the overall genetic heterogeneity model, differences in performance arise. In particular, MDR implemented with CV is significantly better at detecting models that contribute at least 50% to the overall genetic heterogeneity model. There is not, however, a significant difference in the ability of the two methods to detect models that contribute at most 50% to the overall model. This can be attributed primarily to the extremely poor performance of both methods in regard to detecting the less prevalent model.

When the inclusion of false positives into the model predicted by MDR was considered, it was found that MDR implemented with CV is far better than MDR implemented with 3WS at finding exactly one of the two models contributing to the overall genetic heterogeneity model without including any additional loci. The average final model size for MDR implemented with 3WS was about twice that of MDR implemented with CV. This was expected based on previous findings [19] and was one of the main reasons we initially hypothesized that MDR implemented with 3WS would better detect heterogeneity models. Unfortunately, the additional loci included in the final model by MDR implemented with 3WS were not the hard-to-detect disease loci contributing to the heterogeneity model but were instead false positives.

Ultimately, MDR does not appear to be able to effectively detect models exhibiting genetic heterogeneity regardless of the internal model validation method used. Therefore, some other approach must be developed to find this type of model. Ritchie et al. [21] suggested using either cluster analysis or recursive partitioning to confront the challenge presented by genetic heterogeneity. The cluster analysis approach is based on the idea that genetic heterogeneity results from groups of individuals within a population who have different genetic backgrounds. If these groups could be identified prior to looking for associations, then MDR could be run on the groups separately under the assumption that within each group there is only one underlying disease model (and consequently no heterogeneity). Whether using classification trees or cluster analysis, grouping individuals based on a shared genetic background before attempting to identify gene associations seems to be a reasonable direction for further research into finding genetic heterogeneity models. These results highlight the importance of continued development to improve the performance of MDR in the case of heterogeneity, and motivate the use of other approaches if genetic heterogeneity is expected to play a role in the disease etiology.

## Abbreviations

MDR: Multifactor dimensionality reduction; CV: Cross-validation; 3WS: three-way split.

## Competing interests

The authors declare that they have no competing interests.

## Authors’ contributions

JG and HS performed the analysis, helped design the study, and drafted the manuscript. DMR and AMR helped design the study and edited the manuscript. All the authors approved the final version of the manuscript.
